# CGRP and PACAP-38 play an important role in diagnosing pediatric migraine

**DOI:** 10.1186/s10194-022-01435-7

**Published:** 2022-06-13

**Authors:** Junhui Liu, Guan Wang, Yuan Dan, Xinjie Liu

**Affiliations:** Department of Pediatrics, Qilu Hospital of Shandong University, No.107 West Wenhua Road, Jinan, 250012 Shandong Province China

**Keywords:** Pediatric migraine, Pituitary adenylate cyclase-activating polypeptide-38, Calcitonin gene-related peptide, ROC curve, Logistic regression, Diagnostic value

## Abstract

**Background:**

An increasing number of studies have suggested that the important role of vasoactive peptides, such as pituitary adenylate cyclase-activating polypeptide-38 (PACAP-38) and calcitonin gene-related peptide (CGRP), in the pathophysiology of migraine seems undeniable in adults, but studies in pediatric migraine patients remain scarce. We prospectively investigated CGRP and PACAP-38 plasma levels in children with migraine during ictal and interictal periods and compared the results between migraine patients with aura and without aura. We were the first to explore the diagnostic value of a combination of CGRP and PACAP-38.

**Methods:**

Seventy-six migraine patients aged 4–18 years and seventy-seven age-matched healthy children were included in the study. Plasma vasoactive peptides were measured using the enzyme-linked immunosorbent assay (ELISA). Differences and correlations of groups were analyzed using the independent samples t-test, analysis of variance (ANOVA), Mann-Whitney U test, and multiple linear regression. We also performed logistic regression and receiver operating characteristic curve (ROC) analyses to evaluate the diagnostic value of CGRP and PACAP-38 in pediatric migraine.

**Results:**

PACAP-38 and CGRP levels in migraine patients during the ictal and interictal periods were higher than those in controls (*p* < 0.001). PACAP-38 and CGRP levels in migraine patients with aura and without aura were higher than those in controls (*p* < 0.001). PACAP-38 and CGRP were independent risk factors in diagnosing pediatric migraine (adjusted OR (PACAP-38) =1.331, 95% CI: 1.177–1.506, *p* < 0.001; adjusted OR (CGRP) = 1.113, 95% CI: 1.064–1.165, *p* < 0.001). Area Under Curve (AUC) comparison: Combination (0.926) > CGRP (0.869) > PACAP-38 (0.867).

**Conclusions:**

Our study found almost the same changes in CGRP and PACAP levels in pediatric migraine, suggesting that CGRP and PACAP-38 may work together to play an integral role in pediatric migraine. Higher CGRP levels were found in the ictal phase than in the interictal phase and with aura group than without aura group, indicating that CGRP may take part in the formation of pain and aura. Moreover, ROC and logistic regression analyses suggested that CGRP and PACAP-38 are good indicators to diagnose pediatric migraine, and the combination of CGRP and PACAP-38 was valuable in diagnosing pediatric migraine and differentiating pediatric migraine from non-migraine headaches.

**Trial registration:**

The study has been registered at the Chinese Clinical Trial Registry (ChiCTR2100043157).

## Introduction

Migraine is a neurovascular disorder that is common and multifactorial. It is a major cause of disability globally, but the medical literature and studies on the subject have been scarce [1]. Especially in children and adolescents, the diagnosis and treatments of migraine are largely ignored. Migraine is a common headache disorder in children and adolescents [2]. Headache disorders were one of the top ten causes of global DALYs (disability-adjusted life-years) for adolescents in 2019. Headache disorders were the second leading cause for individuals aged 10–24 years, which was just lower than road injuries [3]. A study of the prevalence of migraine in Taiwan suggested that the prevalence was 4.8%, 7.1%, and 8.4% for individuals aged 13, 14, and 15 years, respectively [4]. Many studies have also suggested that the prevalence of migraine increases with increasing age [4, 5] and is consistently higher for girls than boys [4–6]. Migraine can cause absence from school and impaired performance in homework and activities, which result in significant disability in the lives of children and adolescents.

Currently, the diagnosis of migraine follows the third edition of the International Classification of Headache Disorders (ICHD-3), but there are many differences in symptoms between adults and children. Although unilateral location is frequently described in adult patients, the typical headaches in children and adolescents tend to be more bilateral [7]. Another study suggested that younger children have a shorter history of this disease, a reduced frequency of attacks, and a shorter duration of episodes than older children [8]. The diagnosis of migraine is more dependent on the description of the clinical manifestation after normal imaging and laboratory examinations have ruled out other secondary headaches. The difficulty of diagnosing migraine in children is undoubtedly increased by the fact that the child’s memory and description of the symptoms of the headache attack are unclear.

Migraine is defined as a strong genetic component and involves the activation of trigeminal pain pathways. Currently, cortical spreading depression (CSD) pathogenesis is highly valued [9, 10] and has been reported to activate trigeminal and parasympathetic pathways [10]. The activation of the trigeminal ganglion causes the release of these vasoactive peptides, particularly calcitonin gene-related peptide (CGRP) and pituitary adenylate cyclase-activating polypeptide-38 (PACAP-38), from sensory nerves terminals and thus these vasoactive peptides are implicated in pain pathways [11, 12]. Many studies have reported that the plasma levels of CGRP and PACAP-38 in migraine patients are higher than those in healthy people [13–17]. Another clinical trial involves infusing PACAP-38 into healthy participants which also confirmed that the elevation of plasma PACAP-38 can cause migraine attacks [18]. Furthermore, CGRP receptor antagonists and monoclonal antibodies toward CGRP and CGRP receptors showed a positive relief of acute and chronic migraine in clinical trials [19–24].

To date, literature has provided contradictory findings on the association between plasma CGRP, PACAP-38 levels, and pediatric migraine. V Gallai et al. found that CGRP levels in pediatric migraine patients with or without aura were significantly increased during attacks compared with the interictal period [25]. However, Fatma Hanci et al. found that PACAP-38 levels in both ictal and interictal plasma were higher in migraine children without aura than in controls, but CGRP remained unchanged [26]. It remains to be explored whether PACAP-38 and CGRP work in pediatric migraine as a result of the contradictory findings. In addition, there is little literature on whether plasma CGRP and PACAP-38 levels can improve the diagnostic accuracy of migraine in children.

This study prospectively investigated plasma levels of CGRP and PACAP-38 in children with migraine. We compared the plasma CGRP and PACAP-38 levels in patients with age-matched healthy children and analyzed the results according to different subgroups of patients. We also explored the correlation between vasoactive peptides and clinical characteristics of pediatric migraine. Furthermore, we also analyzed the diagnostic value of CGRP, PACAP-38, and the combination of CGRP and PACAP-38 in pediatric migraine.

## Materials and methods

### Study participants

**Fig. 1:**
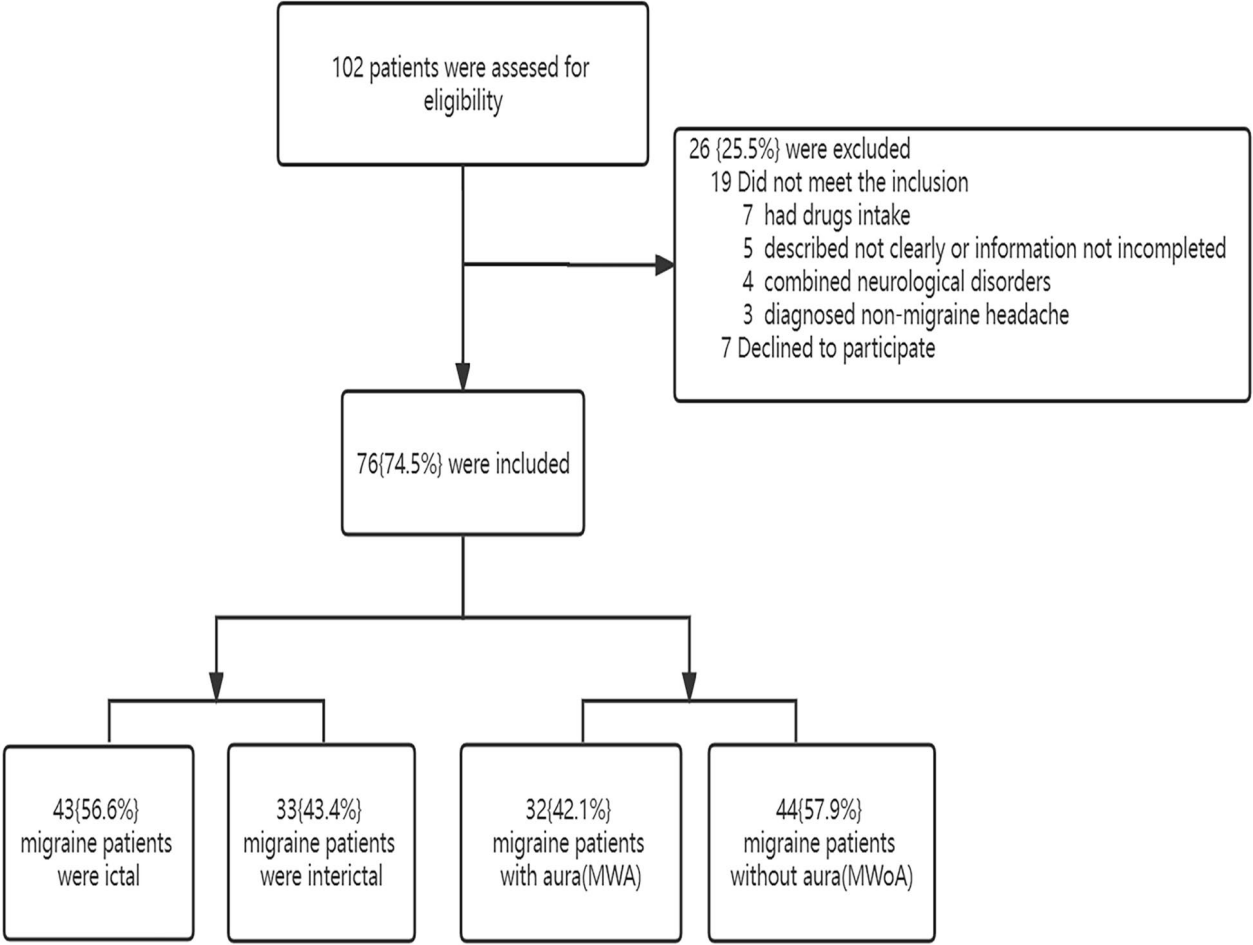
Flowchart indicating the number of excluded patients and reasons for the exclusion

76 children with migraine visiting the pediatric neurology clinic at Qilu Hospital of Shandong University and 77 matched healthy children were enrolled in the study. Inclusion criteria of migraine patients include ages 4 to18 years, a diagnosis of migraine with or without aura defined by the International Classification of Headache Disorders, 3rd Edition [27], and requiring two or more neurologists to make a diagnosis. Exclusion criteria of migraine patients include any analgesic intake for a minimum of 2 months, secondary headaches, mental disorders, congenital disorders, and other major organ disorders. For the controls, we recruited healthy children matched by sex and age. A general physical and neurological examination was required for all participants (Fig. 1).

### Demographic and clinical characteristics

Data collected include age, sex, age of onset, duration of attacks, frequency, course, visual analog scale (VAS) score, a family history of headache, food-related attacks (specific to ice, cheese, chocolate, or monosodium glutamate), sleep quality and sports activities, anxiety and depression. The course of migraine is calculated from the first attack of migraine to the blood sample collection, presented in months. The duration of attacks of migraine is defined as the mean duration per attack per patient, calculated from the beginning to the end of a migraine attack, presented in hours. VAS score (0–10 score) represents the severity of migraine per patient, the higher the score, the heavier the pain. The frequency of migraine is the number of attacks per month. First headache attack age is defined as the age when the patient was firstly attacked by a migraine. The clinical manifestations of migraine were recorded, including the presence of throbbing headache, pain sites, concomitant symptoms, such as nausea, vomiting, photophobia, phonophobia, abdominal pain, ophthalmalgia, and dizziness, and aura symptoms. Visual aura includes flashing, bright spots, dark spots, blurred vision, etc. Sensory aura includes paresthesia in the form of pins and needles and numbness of one side of the body, face or tongue. Motor aura mainly refers to reversible motor weakness.

### Procedures

First, participants were required to fill out the questionnaire, which consisted of participants’ basic information and medical records. When participants were younger than 6 years old, the questionnaire was answered by their guardians. Some foods (specifically ice, cheese, chocolate, or monosodium glutamate), and strenuous activity may induce migraine attacks, therefore, fasting for 8 h and resting for 10 minutes in a seated position were required before obtaining blood. Blood collected during the ictal period was within 8 hours of the onset of migraine. The blood collected during the interictal period required no migraine attack for 24 hours before and after the blood sample collection. Blood was taken from the left or right antecubital vein.

### Measurements

Blood collected was centrifuged for 15 minutes at 4°Cand 3000 rpm (Hangzhou Allsheng Instruments Co., Ltd., Hangzhou, China). The supernatant was stored at − 80°Cuntil assayed. All CGRP and PACAP-38 plasma concentrations in the samples were measured by enzyme-linked immunosorbent assay kits (Jiangsu Meimian Co., Ltd., Jiangsu, China). The detection limits are 0.1 pg/ml for CGRP, 0.1 ng/ml for PACAP-38. Assay protocols were carried out according to the manufacturer’s instructions and were available in duplicate. Briefly, 50 ul of different concentrations of CGRP and PACAP-38 standards were added to their standard wells. Next, sample diluent 40 ul and sample 10 ul were added to sample wells. Set up one sub well per sample. 100 ul of HRP was added to each well, and the 96-well plates were incubated at 37 °C for 60 minutes. Subsequently, washed the plates five times with the wash buffer. Then added 50 ul each of substrate solution A and substrate solution B to each well. The 96-well plates were avoided light and incubated for 15 minutes. Finally, added 50 ul of stop solution to each well, and immediately measured optical density at 450 nm using a microplate reader (Tecan Trading AG, Switzerland). Optical density curves were obtained using standards with determined CGRP and PACAP-38 concentrations.

### Statistical analysis

Statistical analysis was performed using SPSS for Windows (version 27.0, SPSS Inc., Chicago, IL, USA). The Shapiro-Wilk test and Kolmogorov-Smirnov test were used to assess normality differences between or among groups. The data are expressed as the mean ± SD (Standard Deviation) and median ± IQR (interquartile range). Independent samples t-test, ANOVA, and Mann-Whitney U test were used to analyze the differences between the groups. Multiple linear regression was used to analyze the correlation between clinical characteristics and vasoactive peptides. Logistic regression analysis was performed to analyze the association between vasoactive peptides and the diagnosis of pediatric migraine. We performed a ROC analysis, to assess the value of vasoactive peptides in the diagnosis of pediatric migraine. Bilateral *p* < 0.05 was regarded as statistically significant.

## Results

### Study population

We collected plasma samples of participants aged 4–18 years from 76 children with migraine (35 boys, mean age 10.38 ± 3.73 years) and 77 controls (47 boys, mean age 9.46 ± 3.91 years). There was no difference in the mean ages between migraine group and control group (*p* = 0.141). The mean values of course (2.00 ± 8.33 months), duration attacks (2.41 ± 2.86 hours), VAS score (5.11 ± 2.25 scores), and frequency (10.00 ± 11.00 times/month) are shown in Table 1. There were 43 migraine patients in the ictal phase when blood was collected, 32 migraine patients with aura, and 55 migraine patients with concomitant symptoms when headache occurred. 19, 12, and 7 migraine patients experienced visual aura, sensory aura, and motor aura, respectively. The top three concomitant symptoms were nausea (37, 49%), dizziness (28, 37%), and vomiting (24, 32%). Migraine in children is mainly localized over frontal, parietal, and unilateral temporal regions (Table 1).

**Table 1:** Demographic data and comparisons between the migraine group and control group

Characteristics	Migraine	Control	***P***-value
**Number**	76	77	
Age (years)	10.38 ± 3.73	9.46 ± 3.91	0.141
Gender (male)	35 (46%)	47 (61%)	0.064
**Clinical Characteristics**			
Course (months)	2.00 ± 8.33		
Duration attacks (hours)	2.41 ± 2.86		
VAS score	5.11 ± 2.25		
Frequency (times/month)	10.00 ± 11.00		
Ictal phase	43 (57%)		
Aura	32 (42%)		
Concomitant symptoms	55 (72%)		
**Aura**			
Visual aura	19 (25%)		
Sensory aura	12 (16%)		
Motor aura	7 (9%)		
**Concomitant symptoms**			
Nausea	37 (49%)		
Vomiting	24 (32%)		
Photophobia	18 (24%)		
Phonophobia	12 (16%)		
Dizziness	28 (37%)		
Ophthalmalgia	11 (14%)		
Abdominal pain	14 (18%)		
**Pain site**			
Unilateral temporal	21 (28%)		
Bilateral temporal	13 (17%)		
Frontal	28 (37%)		
Parietal	23 (30%)		
Occipital	10 (13%)		

When blood samples were collected, 43 migraine patients had headache attacks (ictal phase), and 33 migraine patients had no headache attacks (interictal phase). We found an inapparent difference between the mean ages of the ictal phase (11.16 ± 3.51 years) and the interictal phase (9.36 ± 3.81 years, *p* = 0.036). There was no significant difference in the mean values of course, duration of attacks, VAS score, frequency, aura, or concomitant symptoms between migraine patients in the ictal phase and in the interictal phase (Table 2). There were 32 patients with migraine with aura groups (MWA) and 44 patients with migraine without aura groups (MWoA). There was no significant difference in the mean values of course, VAS score, frequency, ictal phase, or concomitant symptoms between migraine patients with aura and without aura (Table 3).

**Table 2:** Demographic data and comparisons between the ictal phase and interictal phase of migraine patients

Characteristics	Ictal phase	Interictal phase	***P***-value
Number	43	33	
Age (years)	11.16 ± 3.51	9.36 ± 3.81	0.036
Gender (male)	19 (44%)	16 (48%)	0.714
**Clinical Characteristics**			
Course (months)	2.00 ± 5.42	1.00 ± 11.42	0.397
Duration attacks (hours)	2.86 ± 2.94	1.83 ± 2.69	0.121
VAS score	5.42 ± 2.30	4.71 ± 2.15	0.177
Frequency (times/month)	15.00 ± 17.50	8.00 ± 9.50	0.268
Aura	18 (42%)	14 (42%)	0.961
Concomitant symptoms	34 (79%)	21 (64%)	0.149

**Table 3:** Demographic data and comparisons between aura group and without aura group of migraine patients

Characteristics	Aura	Without aura	*P*-value
Number	32	44	
Age (years)	10.94 ± 3.82	9.98 ± 3.66	0.271
Gender (male)	11 (34%)	24 (55%)	0.084
**Clinical Characteristics**			
Course (months)	1.00 ± 10.75	2.00 ± 4.83	0.313
Duration attacks (hours)	2.44 ± 2.81	2.39 ± 2.94	0.937
VAS score	5.28 ± 2.20	4.99 ± 2.31	0.580
Frequency (times/month)	13.00 ± 12.00	10.00 ± 13.50	0.073
Ictal phase	18 (56%)	25 (57%)	0.961
Concomitant symptoms	25 (78%)	30 (68%)	0.345

### Plasma CGRP levels in pediatric migraine

**Fig. 2:**
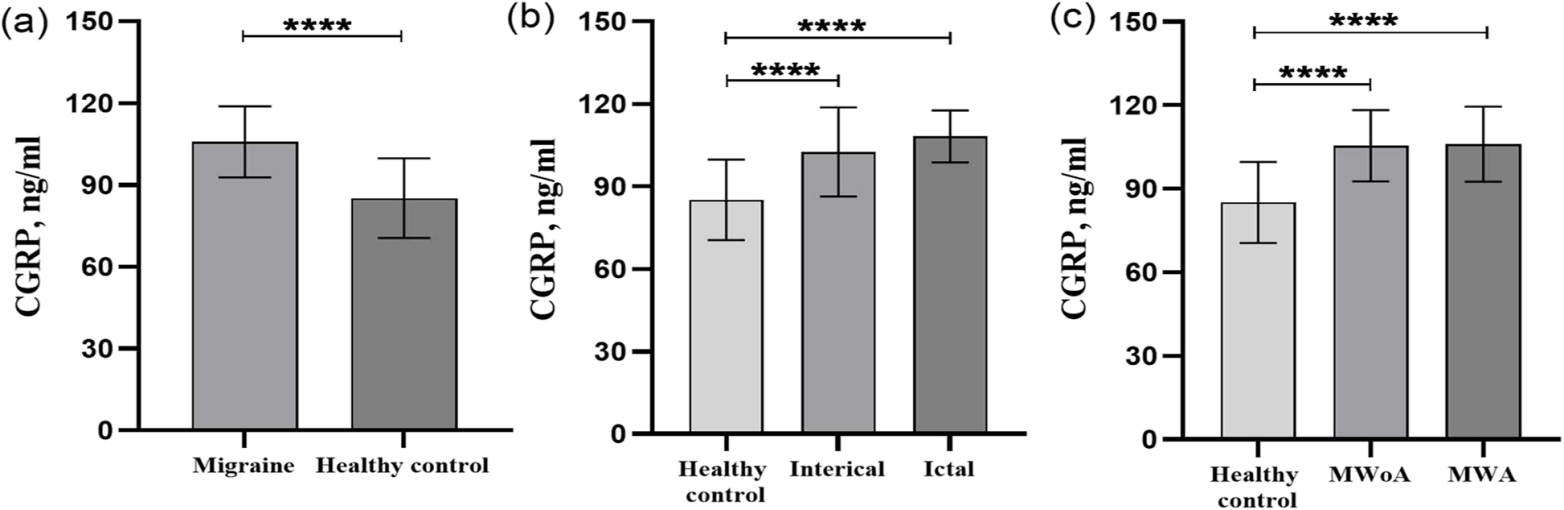
Calcitonin gene-related peptide (CGRP) plasma levels in different subgroups of migraine group and control group. Plasma levels of CGRP in the migraine and control groups, ictal and interictal groups, MWA (migraine with aura), and MWoA (migraine without aura) (**a, b, c**). ****p < 0.001

We investigated the plasma CGRP concentration of the migraine group and control group and the CGRP levels of migraine group were significantly higher than those of control group (Fig. 2a) (CGRP(m) = 105.75 ± 13.01 pg/ml, CGRP(c) = 85.14 ± 14.58 pg/ml, *p* < 0.001). There was a significantly higher plasma concentration of CGRP in the ictal phase and the interictal phase of migraine group compared to the control group (Fig. 2b) (CGRP(ictal) = 108.19 ± 9.40 pg/ml, CGRP(interictal) = 102.56 ± 16.19 pg/ml, CGRP(c) = 85.14 ± 14.58 pg/ml, *p* < 0.001). Although there was a tendency for plasma CGRP levels to be elevated in migraine patients in the ictal period compared to the interictal period, no significant difference in plasma CGRP levels were found between the two groups. Furthermore, we found that the CGRP levels of the migraine with aura and without aura groups were notably higher than those of the control group (Fig. 2c) (CGRP(MWA) = 106.10 ± 13.48 pg/ml, CGRP(MWoA) = 105.49 ± 12.81 pg/ml, CGRP(c) = 85.14 ± 14.58 pg/ml, *p* < 0.001). However, there was no significant difference in plasma CGRP levels between migraine with aura and without aura groups.

### Plasma PACAP-38 levels in pediatric migraine

**Fig. 3:**
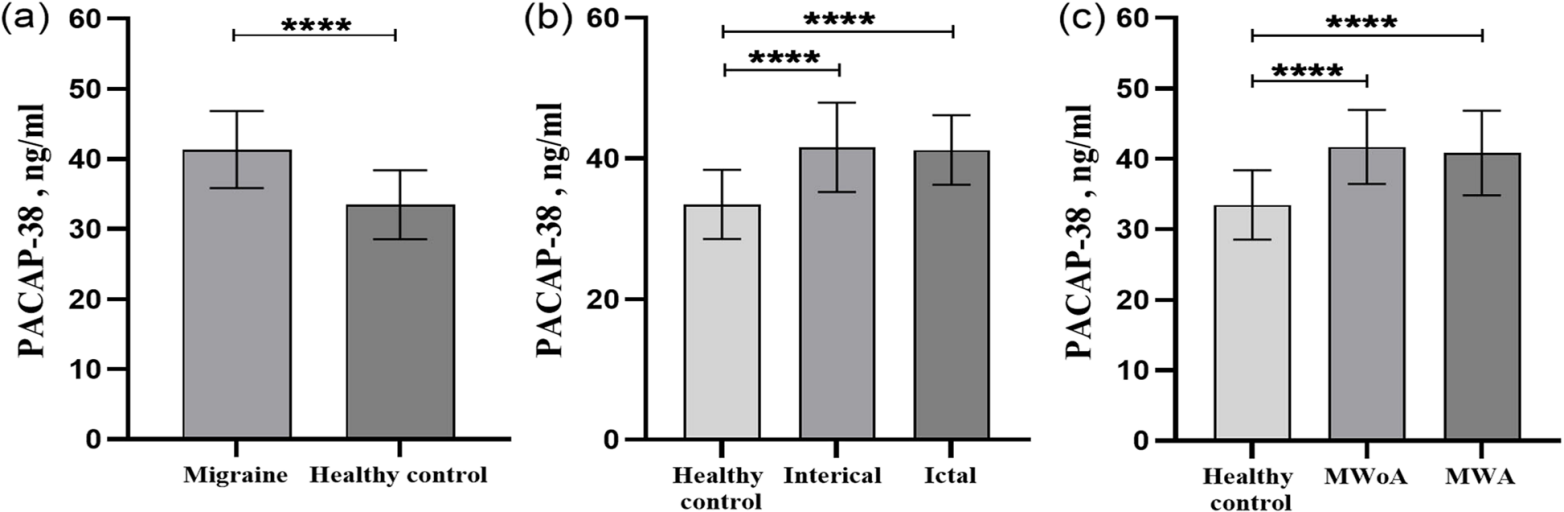
Pituitary adenylate cyclase-activating polypeptide-38 (PACAP-38) plasma levels in different subgroups of migraine and control groups. Plasma levels of PACAP-38 in the migraine and control groups, ictal and interictal groups, MWA (migraine with aura), and MWoA (migraine without aura) (**a, b, c**). ****p < 0.001

We found that the PACAP-38 levels of migraine group were notably higher than those of control group (Fig. 3a) (PACAP-38(m) = 41.32 ± 5.49 ng/ml, PACAP-38(c) = 33.44 ± 4.92 ng/ml, *p* < 0.001). The PACAP-38 levels during the ictal period and interictal period were significantly higher than those in the control group (Fig. 3b) (PACAP-38(ictal) = 41.19 ± 4.95 ng/ml, PACAP-38(interictal) = 41.58 ± 6.35 ng/ml, PACAP-38(c) = 33.44 ± 4.92 ng/ml, *p* < 0.001). However, we did not find a difference in PACAP-38 levels between the ictal period and the interictal period. There were higher PACAP-38 levels in the migraine with aura and without aura groups than in the control group (Fig. 3c) (PACAP-38(MWA) = 40.83 ± 5.99 ng/ml, PACAP-38(MWoA) = 41.97 ± 5.26 ng/ml, PACAP-38(c) = 33.44 ± 4.92 ng/ml, *p* < 0.001). However, no significant difference was observed in plasma PACAP-38 levels between migraine with aura and migraine without aura.

### Plasma CGRP and PACAP-38 levels in pediatric migraine with aura

**Fig. 4:**
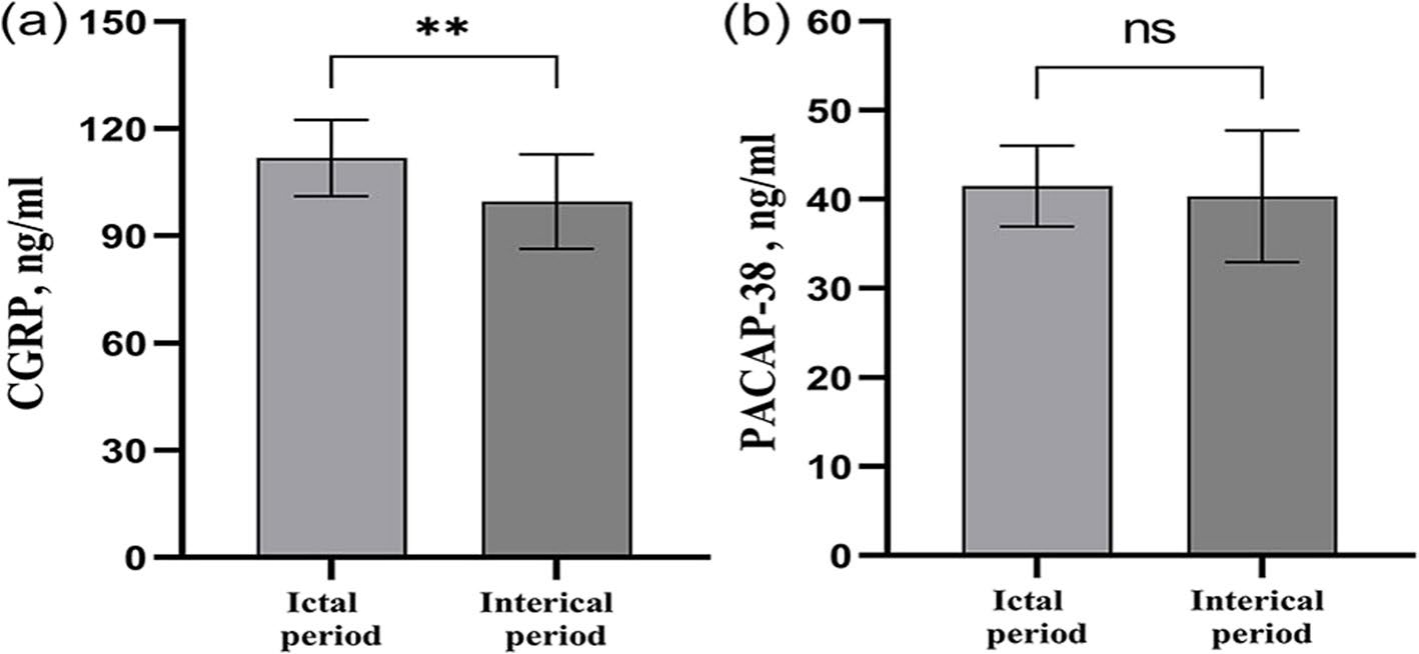
CGRP and PACAP-38 plasma levels in ictal and interictal groups of migraine with aura groups. CGRP plasma levels in ictal and interictal groups of migraine with aura groups (**a**). PACAP-38 plasma levels in ictal and interictal groups of migraine with aura groups (**b**). CGRP: Calcitonin gene-related peptide, PACAP-38: pituitary adenylate cyclase-activating polypeptide-38. **p < 0.01, ns: nonsignificant

Migraine with aura can lead to more discomfort in patients, which is worthy of our attention. Therefore, we analyzed the migraine with aura groups separately. Patients with migraine with aura have significantly higher plasma CGRP levels during the ictal period than during the interictal period (Fig. 4a) (CGRP(ictal) = 111.76 ± 10.74 pg/ml, CGRP(interictal) = 99.55 ± 13.27 pg/ml, *p* = 0.006). There was a tendency for plasma PACAP-38 levels to increase in the ictal period of migraine patients with aura, but no difference was found between the two groups (Fig. 4b) (PACAP-38(ictal) = 41.46 ± 4.56 ng/ml, PACAP-38 (interictal) = 40.32 ± 7.38 ng/ml, *p* > 0.05). Furthermore, we also compared the CGRP and PACAP-38 levels among the visual, sensory, and motor aura groups. There was no significant difference among visual, sensory, and motor aura groups (CGRP(visual) = 109.72 ± 13.24 pg/ml, CGRP(sensory) = 104.20 ± 13.65 pg/ml, CGRP(motor) = 99.84 ± 14.71 pg/ml) *p* = 0.381; PACAP-38(visual) = 40.59 ± 7.41 ng/ml, PACAP-38(visual) = 42.34 ± 3.95 ng/ml, PACAP-38(visual) = 40.88 ± 6.22 ng/ml, *p* = 0.806).

### Plasma CGRP and PACAP-38 levels in pediatric migraine in the ictal phase

**Fig. 5:**
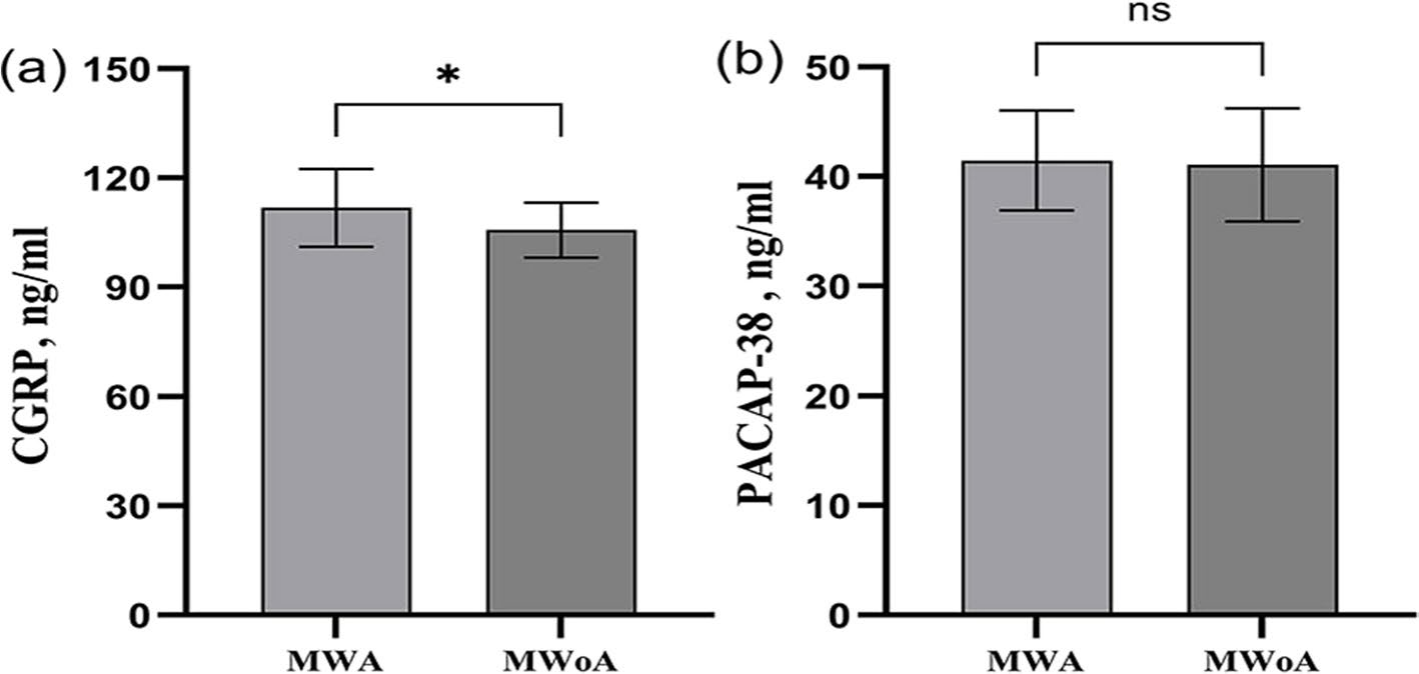
CGRP and PACAP-38 plasma levels in MWA and MWoA groups of migraine in ictal period. CGRP plasma levels in migraine with MWA and MWoA groups of migraine in ictal period (**a**). PACAP-38 plasma levels in migraine with MWA and MWoA groups of migraine in ictal period (**b**). CGRP: Calcitonin gene-related peptide, PACAP-38: pituitary adenylate cyclase-activating polypeptide-38, MWA:migraine with aura, and MWoA: migraine without aura. *p < 0.05, ns: nonsignificant

The ictal phase is another important clinical characteristic of migraine that we should pay more attention to. Therefore, we analyzed the migraine groups in the ictal phase separately. We found that plasma CGRP levels were significantly higher in the migraine patients with aura than without aura during the ictal period (Fig. 5a) (CGRP(MWA) = 111.76 ± 10.74 pg/ml, CGRP(MWoA) = 105.63 ± 7.53 pg/ml, *p* = 0.033). In the patients in the ictal period, there was a tendency for plasma PACPA-38 levels to be elevated in the migraine patients with aura, but no difference was observed between the two groups (Fig. 5b) (PACAP-38(MWA) = 41.46 ± 4.56 ng/ml, PACAP-38(MWoA) = 41.06 ± 5.16 ng/ml, *p* > 0.05).

### The correlation of CGRP and PACAP-38 levels with clinical characteristics

Univariate regression analysis showed that the plasma PACAP-38 levels correlated to the course, duration of attacks, headache frequency, and vomiting, and the plasma CGRP levels were relative to the frequency, nausea, and bilateral temporal location of migraine (Table 4). We selected the clinical characteristics with *P*-value less than 0.2 into the multiple linear regression analysis. It revealed that the plasma PACAP-38 level correlated to the course (< 6 months) (*p* = 0.041) and duration of attacks (< 2 hours) (*p* = 0.001) by controlling VAS score, frequency, vomiting, photophobia, abdominal pain (Table 5). It also showed that the plasma CGRP level correlated to duration attacks (> 6 hours) (*p* = 0.006), frequency (< 15 times/month) (*p* = 0.050), and nausea (*p* = 0.021) by controlling bilateral temporal part and frequency (Table 6).

**Table 4:** Univariate analysis of the correlation between vasoactive peptides and clinical characteristics

Characteristics	PACAP-38 (ng/ml)	P-value	CGRP (pg/ml)	*P*-value
**Age (years)**		0.503		0.573
0–6	40.65 ± 7.74		102.44 ± 13.35	
6–12	40.82 ± 4.79		106.25 ± 14.10	
12–18	42.33 ± 5.05		106.82 ± 11.48	
Gender		0.277		0.579
Man	42.07 ± 4.71		106.65 ± 10.71	
Women	40.69 ± 6.06		104.98 ± 14.79	
**Course (months)**		0.017*		0.582
0–6	42.33 ± 5.04		106.82 ± 11.48	
6–12	40.38 ± 5.43		104.11 ± 13.57	
12–18	42.27 ± 4.33		111.38 ± 11.86	
18–24	33.03 ± 3.79		104.43 ± 10.56	
> 24	46.23 ± 3.42		108.74 ± 7.39	
**Duration attacks (hours)**		0.004**		0.063
0–2	41.82 ± 4.81		105.91 ± 13.04	
2–4	39.32 ± 5.46		102.46 ± 13.25	
4–6	43.43 ± 3.22		109.08 ± 5.93	
> 6	42.31 ± 5.50		105.16 ± 9.13	
**VAS score**		0.092		0.726
0–3	46.18 ± 3.75		110.87 ± 7.39	
4–6	41.93 ± 6.82		105.15 ± 17.25	
7–10	40.75 ± 5.23		105.94 ± 10.84	
**Frequency (times/month)**		0.005**		0.011*
0–15	45.04 ± 3.82		113.63 ± 14.16	
15–30	42.07 ± 4.87		107.89 ± 12.44	
30–60	40.36 ± 4.18		100.42 ± 11.23	
**Aura**				
Visual aura	40.10 ± 6.84	0.266	108.32 ± 13.36	0.322
Sensory aura	41.01 ± 4.84	0.786	103.29 ± 13.34	0.362
Motor aura	38.90 ± 5.13	0.222	102.56 ± 15.90	0.500
**Concomitant symptoms**				
Nausea	40.94 ± 6.23	0.552	109.28 ± 12.55	0.020*
Vomiting	43.49 ± 4.68	0.019*	107.57 ± 13.42	0.411
Photophobia	43.03 ± 7.57	0.133	107.75 ± 15.72	0.459
Phonophobia	42.04 ± 8.75	0.625	104.04 ± 15.12	0.624
Dizziness	40.97 ± 5.98	0.671	107.42 ± 13.38	0.396
Ophthalmalgia	41.51 ± 4.50	0.904	109.30 ± 5.73	0.330
Abdominal pain	39.14 ± 6.70	0.100	102.85 ± 13.17	0.360
**Pain site**				
Unilateral temporal	41.85 ± 4.93	0.611	105.91 ± 11.35	0.948
Bilateral temporal	42.41 ± 3.94	0.437	112.43 ± 12.64	0.041*
Frontal	40.49 ± 6.28	0.312	103.97 ± 13.56	0.365
Parietal	40.03 ± 4.42	0.177	104.48 ± 11.86	0.578
Occipital	42.18 ± 5.03	0.608	107.20 ± 10.26	0.708

*p < 0.05; **p < 0.01

Clinical Characteristics with P less than 0.2 were included in the multivariate analysis of the correlation between vasoactive peptides and clinical characteristics.

**Table 5:** Multivariate analysis of the correlation between PACAP-38 levels and clinical characteristics

Characteristics	β	95%CI	*P*-value
**Course (months)**			
0–6	−2.721	(−5.326, − 0.116)	0.041*
**Duration attacks (hours)**			
0–2	−4.045	(−6.384, −1.705)	0.001**

*p < 0.05; **p < 0.01

**Table 6:** Multivariate analysis of the correlation between CGRP levels and clinical characteristics

Characteristics	β	95%CI	*P*-value
**Duration attacks (hours)**			
> 6	9.785	(2.921, 16.649)	0.006**
**Frequency (times/month)**			
0–15	5.680	(0.002, 11.357)	0.050*
**Concomitant symptoms**			
Nausea	6.527	(1.032, 12.022)	0.021*

*p < 0.05; **p < 0.01

### The association of CGRP, PACAP-38, and the diagnosis of pediatric migraine

The association of CGRP, PACAP-38, demographic variables, and the diagnosis of pediatric migraine was analyzed by binary logistic regression. Demographic variables include age and gender. ANOVA analysis showed that CGRP and PACAP-38 levels are associated with the diagnosis of pediatric migraine (p(CGRP) = 0.012, p(PACAP-38) < 0.001), while age and gender were not associated with the diagnosis of migraine in children (age; *p* = 0.23; gender; *p* = 0.064). Therefore, CGRP and PACAP-38 plasma levels were included in multiple logistic regression analyses, showing that PACAP-38 and CGRP were independent risk factors in the diagnosis of pediatric migraine (adjusted OR (PACAP-38) = 1.331, 95% CI: 1.177–1.506, *p* < 0.001; adjusted OR (CGRP) = 1.113, 95% CI: 1.064–1.165, *p* < 0.001), which indicated that every 10 unit increase in plasma CGRP levels and PACAP-38 levels is associated with 11.13 times and 13.31 times increase in the diagnosis rate of pediatric migraine (Table 7).

**Table 7:** Multivariable logistic regression of association between CGRP, PACAP-38 and the diagnosis of pediatric migraine

	OR	95%CI	P-value
CGRP	1.113	1.064–1.165	< 0.001
PACAP-38	1.331	1.177–1.506	< 0.001

### The diagnostic value of CGRP and PACAP-38 in pediatric migraine

**Fig. 6:**
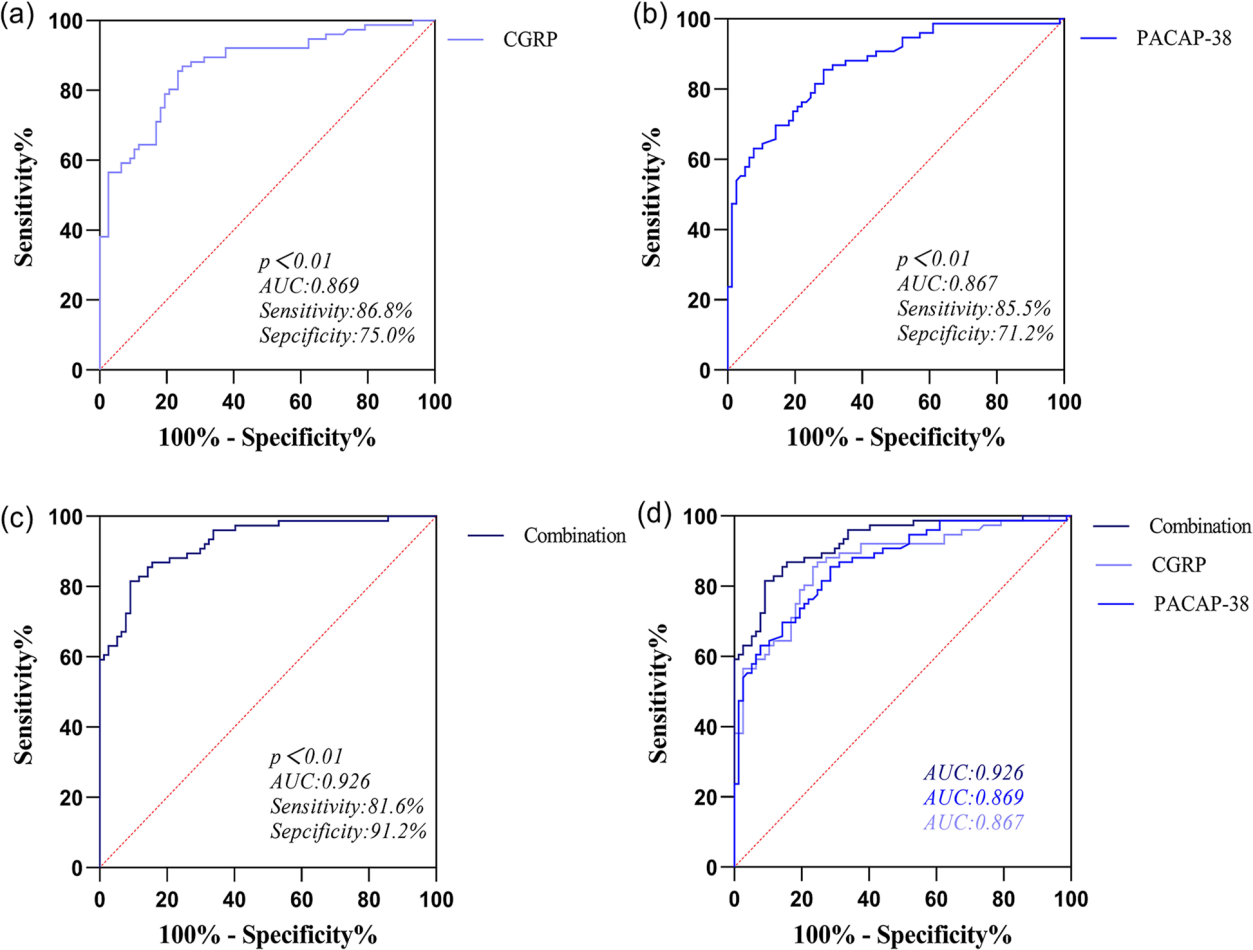
The ROC curve of CGRP, PACAP-38 and the combination in diagnosing pediatric migraine. The ROC curve of CGRP in diagnosing pediatric migraine (**a**). The ROC curve of the PACAP-38 in diagnosing pediatric migraine (**b**). The ROC curve of the combination in diagnosing pediatric migraine (**c**). The total ROC curve of CGRP, PACAP-38 and the combination (**d**). AUC: area under the curve, CGRP: calcitonin gene-related peptide, PACAP-38: pituitary adenylate cyclase-activating polypeptide-38, Combination: the predictive probability of the combination of CGRP and PACAP-38 in diagnosing pediatric migraine

Figure 6 showed the analysis results of the ROC curve of CGRP, PACAP-38, and the combination of CGRP and PACAP-38 in diagnosing pediatric migraine. We found the combination of CGRP and PACAP-38 had the best diagnostic value in pediatric migraine (AUC: Combination (0.926) vs CGRP (0.869) vs PACAP-38 (0.867)) and had the highest specificity (specificity: Combination (90.91%) vs CGRP (76.62%) vs PACAP-38 (71.43%)). But the sensitivity of the combination of CGRP and PACAP-38 was slightly lower than CGRP and PACAP-38 alone (sensitivity: CGRP (85.53%) vs PACAP-38 (85.53%) vs Combination (81.58%)). The cut-off points of PACAP-38, CGRP, and the combined index were 36.57 ng/ml, 94.29 pg/ml, and 0.55 points, respectively (Table 8).

**Table 8:** ROC analysis of the diagnostic value of CGRP, PACAP-38, and their combination for pediatric migraine

	AUC	95%CI	Sensitivity%	Specificity%	+LR	-LR	Youden Index	Cut-Off
CGRP	0.869	0.812–0.925	85.53	76.62	3.659	0.189	0.621	94.29
PACAP-38	0.867	0.810–0.922	85.53	71.43	2.993	0.203	0.570	36.57
Combination	0.926	0.886–0.966	81.58	90.91	8.974	0.203	0.725	0.55

Abbreviations: *+LR* Positive likelihood ratio, *−LR* Negative likelihood ratio

## Discussion

Our findings indicated that plasma CGRP and PACAP-38 levels in children with migraine were significantly higher than those in control group. Similarly, Fan PC et al. have reported pediatric patients with migraine had higher plasma CGRP levels than non-headache children [28]. We found that plasma CGRP and PACAP-38 levels were higher in children with migraine than in control group during both attacks and non-attacks, and the plasma CGRP and PACAP-38 levels were elevated in the migraine with and without aura group than in control group, same results as for some adults with migraine [29, 30]. Hanci F et al. found higher plasma PACAP-38 levels, but not CGRP levels in children with migraine without aura than in healthy children during both attacks and non-attacks [26]. P-C Fan et al. described that CGRP levels were higher in the ictal phase than in the controls, but no difference was found between the interictal phase and healthy children [28]. These findings indirectly suggest that CGRP and PACAP-38 play an important role in the pathogenesis of migraine in children. The mechanisms of CGRP mediating pediatric migraine mainly are involved in the vasodilation theory [31, 32], neurogenic inflammation [33, 34], peripheral and central sensitization [35, 36], and cortical diffusion inhibition diffusion (CSD) [37–39], and nitric oxide generation. However, CGRP alone cannot explain all clinical manifestations of migraine and may have a cooperative action with other peptides in inducing migraine attacks, such as PACAP [40]. Interestingly, PACAP has several similar actions with CGRP in inducing migraine attacks, and PACAP also triggers CGRP release in the TNC, which researchers speculate is a potential epistatic modulator of CGRP in migraine [40], suggesting that CGRP and PACAP might work together to induce migraine attacks, which is supported at a molecular level by the fact that CGRP and PACAP receptors share a RAMP1 (receptor activity modifying protein 1) subunit [14].

Because migraine attacks with aura symptoms and migraine in the ictal period can cause more discomfort for patients, we paid more attention to groups with aura symptoms and groups in the ictal period. The findings indicated that in the migraine with aura group, CGRP levels in the ictal phase were higher than those in the interictal phase, suggesting that CGRP may be involved in the formation of pain in children with migraine [36, 41] since pain is perceived by patients during migraine attacks. CGRP can sensitize peripheral nociceptors and enhance central nervous system sensory input to heighten pain perception. CGRP is expressed on almost 50% of human trigeminal ganglion (TG) neurons, most of them on unmyelinated nociceptive C-fibers. CGRP receptors are expressed on myelinated A-fibers. When trigeminal neurons are activated, CGRP is released from C-fibers, and act on receptors of A-fibers to stimulate second-order trigeminal nucleus caudalis (TNC) neurons and then relay to the thalamus, ultimately causing central sensitization [42–44]. In migraine in the ictal phase group, we found that plasma CGRP levels in the migraine with aura group were significantly higher than those in the migraine without aura group. Gallai V et al. have reported increased CGRP levels in pediatric migraine with aura compared with migraine without aura in the ictal period, indicating that CGRP may induce aura symptoms of migraine in children [37–39]. Several findings support that CGRP can influence the formation of aura symptoms by mediating the mechanism of CSD. CSD is related to aura symptoms in migraine, which may interact with CGRP. During CSD, more endogenous CGRP could be released, which was revealed by using rat cortical brain slices [37]. CGRP receptor antagonists weakened pial dilation induced by CSD [38, 39] and the magnitude of the CSD effect in vitro [45].

Multiple linear regression analysis showed that plasma CGRP levels correlated to nausea, which is consistent with the existence of CGRP in the enteric nervous system, involved in regulating gastrointestinal motility and secretions [14]. But there were no correlations between CGRP plasma levels and other gastrointestinal symptoms, such as vomiting and abdominal pain. We think lots of factors can take responsibility for gastrointestinal symptoms. For example, some patients have dyspepsia or an irregular diet, such as spicy food, eating too much, and eating food before sleeping, which also can cause gastrointestinal symptoms. These correlations between CGRP, abdominal pain, and vomiting may be influenced by the inability to remove confounding factors due to the limited clinical information available.

Multiple logistic regression showed that CGRP and PACAP-38 are independent risk factors associated with the diagnosis of pediatric migraine. ROC analysis suggested that CGRP and PACAP-38 are of great value in diagnosing migraine in children because of their high AUC, sensitivity, and specificity. Fan PC et al. have reported similar sensitivity, specificity, and positive likelihood ratio of CGRP in diagnosing pediatric migraine [28]. Considering the similar plasma level changes of CGRP and PACAP-38 in our studies and the similar effects in the pathogenesis of migraine discussed in previous studies, we considered the combined diagnostic value of CGRP and PACAP-38. The findings indicated that the combination of CGRP and PACAP-38 had the greatest AUC and specificity compared to CGRP and PACAP-38, suggesting its superior value in diagnosing pediatric migraine and differentiating pediatric migraine from non-migraine. To the best of our knowledge, this is the first study to analyze the diagnostic value of PACAP-38 in pediatric migraine and the first to combine CGRP with PACAP-38 as a combination marker to diagnose migraine in children.

Furthermore, we found that the duration of attacks of some children with migraine was less than 2 hours, which cannot be explained by the selection bias that would be more likely to select children with longer pain episodes. Battistella et al. and Raieli V et al. have reported the shorter duration of headaches in younger children, which can be by active mechanisms (e.g., sleeping), rather than physiological pain mechanisms [8, 46]. Sleeping can alleviate headache attacks, and poor sleep quality might increase the risk of headache attacks. Preschool children, especially those under 6 years old, sleep longer and they are usually more likely to sleep during the day [46]. Therefore, the headache attacks are easily stopped and cause a shorter duration per attack.

And almost half of the children with migraine have aura symptoms and most of them have concomitant symptoms. The VAS score showed that 79% (66) of patients have moderate to severe migraine. Migraine seriously affects the healthy quality of life of children, worthy of attention of the clinicians.

## Conclusion

Our study found significant elevation and almost the same changes in CGRP and PACAP-38 levels in pediatric migraine, which suggested that CGRP and PACAP-38 play an integral role in pediatric migraine and may work together in the pathogenesis of migraine in children. Multiple logistic regression showed that CGRP and PACAP-38 are independent risk factors for pediatric migraine. Moreover, there was a high AUC, the best sensitivity, and a higher OR of CGRP and PACAP-38, suggesting that CGRP and PACAP-38 are good indicators to diagnose pediatric migraine, and the combination of CGRP and PACAP-38 has a great value in diagnosing pediatric migraine and distinguishing migraine and non-migraine headache in terms of the largest AUC and specificity. Higher CGRP levels were found in the ictal phase group than in the interictal phase and in the aura group than in the without aura group, which indicated that CGRP may take part in the formation mechanism of pain and aura. Therefore, in the future, more prospective clinical studies including more patients can explore the sensitivity and specificity of CGRP and PACAP-38 in diagnosing migraines. Furthermore, more basic studies should pay more attention to the association of molecular pathways between CGRP and PACAP-38.

## Limitations

Children younger than 6 years have an unclear memory and description of the symptoms of the migraine attacks. For example, they cannot describe the site or the severity of migraine attacks clearly. If necessary, the description of the guardians of the younger children should be referred. The information we collect (frequency of migraine, presence or absence of aura, etc.) depends on recall of the patients and/or parents. Therefore, recall bias may occur. In collecting the data, we gave the patient sufficient time to recall and combine it with their guardian’s description in order to try to minimize the possibility of bias. In addition, selection bias was inevitable and all researchers preferred to select the more severe and typical cases. With regards this, information collection, data analysis and interpretation of results are the responsibility of different researchers and they were prohibited from discussing the clinical information collected and trial data with each other.

## Data Availability

The datasets used or analyzed during the current study are available from the corresponding author on reasonable request.

## Abbreviations

PACAP-38: Pituitary adenylate cyclase-activating polypeptide-38
CGRP: Calcitonin gene-related peptide
DALYs: Disability-adjusted life-years
ICHD-3: International Classification of Headache Disorders
CSD: Cortical spreading depression
VAS: Visual analog scale
MWA: Migraine with aura
MWoA: Migraine without aura
AUC: Area under the curve
+LR: Positive likelihood ratio
-LR: Negative likelihood ratio
TNC: Trigeminal nucleus caudalis
RAMP1: Receptor activity modifying protein 1
TG: Trigeminal ganglion
